# Surface Modification of Aluminum Nitride to Fabricate Thermally Conductive poly(Butylene Succinate) Nanocomposite

**DOI:** 10.3390/polym11010148

**Published:** 2019-01-16

**Authors:** Zelalem Lule, Jooheon Kim

**Affiliations:** School of Chemical Engineering & Materials Science, Chung-Ang University, Seoul 156-756, Korea; zochernet@gmail.com

**Keywords:** poly(butylene succinate), nanocomposite, thermal conductivity

## Abstract

Biodegradable polymers and their composites are considered promising materials for replacing conventional polymer plastics in various engineering fields. In this study, poly(butylene succinate) (PBS) composites filled with 5% aluminum nitride nanoparticles were successfully fabricated. The aluminum nitride nanoparticles were surface-modified to improve their interaction with the PBS matrix. Field-emission scanning electron microscopy revealed that the nanocomposites with surface-modified nanoparticles had better interface interaction and dispersion in the polymer matrix than those with untreated nanoparticles. The PBS/modified AlN nanocomposites exhibited maximal thermal conductivity enhancement, 63.7%, compared to the neat PBS. In addition, other thermomechanical properties of the PBS nanocomposites were investigated in this study. The nanocomposites also showed a superior storage modulus compared to the neat PBS matrix. In this work, a PBS nanocomposite with suitable thermal conductivity that can be used in various electronic fields was fabricated.

## 1. Introduction

The development of petroleum-based plastic products has brought great benefits to people’s daily life [1]. Currently, the large consumption of these plastics in the packaging, agricultural, and automobile industries has become a great concern for the environment [2]. Therefore, many countries are enacting rules and legislations to regulate the consumption and disposal of plastics made from conventional polymers. As a result, many industrial and academic studies have focused on the development and applications of eco-friendly materials that can replace non-degradable petroleum-based materials [1,2,3,4]. Aliphatic biodegradable polymers, composites, and blends have been the preeminent solution for fabricating eco-friendly materials. Biodegradable polymers include polylactic acid, polycaprolactone, poly(butylene succinate) (PBS), and poly hydroxyalkanoates. PBS is one of the most promising aliphatic polyesters, owing to its low cost, processability, biodegradability, and compostability [4,5].

PBS—a biodegradable thermoplastic polyester—is synthesized via the polycondensation of 1,4-butanediol with succinic acid [2,6]. It has excellent melt processability, as well as thermal and mechanical properties comparable to those of the conventional polyolefin plastic materials, such as polyethylene (PE), polyethylene terephthalate, and polypropylene (PP) [7,8,9]. PBS was first invented by Showa Highpolymer (Japan) in the early 1990s, with both the monomers originating from petrochemical sources [10]. Many researchers and companies have confirmed that 1,4-butanediol and succinic acid can be obtained from biomass sources, which boosts the commercial large-scale production of cheap bio-based PBS [11,12,13]. Therefore, PBS has significant potential to be used as an alternative to petroleum-based plastics such as PE and PP. PBS is currently used in various daily applications, such as for fishing lines, foam cushioning and mulch films in agriculture, and packaging, e.g., bottles, bags, and film. Owing to its ecological and economic advantages, PBS, as an engineering plastic, is likely to have applications in the electronics, automotive, and aerospace industries [14]. However, PBS has inherent shortcomings, such as insufficient stiffness, low thermal conductivity, and low melt strength, that limit its application in various engineering fields [3].

To enhance the properties of the PBS polymer, the fabrication of the nanocomposite via the incorporation of nanoparticles into the matrix is a widely used and effective approach. In previous studies, PBS composites have been prepared using various kinds of fillers, such as inorganic oxides, ceramics, and carbonaceous materials. Huang et al. [6] fabricated TiO_2_-filled PBS nanocomposites using a novel vane extruder. The PBS/TiO_2_ nanocomposites exhibited a high storage modulus, but the crystallization and melting behavior of PBS was not affected. Shih et al. [9] used simple melt blending to fabricate a PBS/multiwall carbon nanotube nanocomposite with improved thermal and mechanical properties. In addition, the mechanical properties of melt-extruded raw silica and silane-treated silica-filled PBS were compared and the results revealed that the PBS/treated silica composite had superior tensile and impact properties [15]. Moreover, in a previous study, we fabricated a PBS/Al_2_O_3_ composite with twice the thermal conductivity of neat PBS, via the addition of 30% treated alumina to the PBS matrix [16].

PBS is one of the promising biopolymers expected to replace conventional polyolefins in the electrical and packaging industries, where thermal conductivity and rigidity are desirable for an appreciable level of thermal conductance in circuit boards, heat exchangers, appliances, and machinery. Among the inorganic fillers, AlN is considered the best candidate for fabricating thermally conductive PBS composites, mainly owing to its attractive properties, such as its high thermal conductivity, high electrical resistivity, non-toxicity, and low cost. Rajeshwari et al. [17,18] fabricated high-density polyethylene (HDPE)/AlN nanocomposites with different volume fractions of AlN. The nanocomposite with 20% AlN loading exhibited high stiffness and a thermal-conductivity enhancement of 40% compared with pure HDPE. Thermal conductivity of 0.638 W m^−1^ k^−1^ was achieved for a PP/AlN composite with the incorporation of 25% micro-size AlN particles [19]. Ma et al. [20] used pure AlN and silane-treated AlN to fabricate a PP/AlN composite. The thermal conductivity of the composite increased to 1.84 W m^−1^ k^−1^ with the addition of 50 vol.% silane-modified AlN. Gu et al. [21] synthesized a thermally conductive AlN/low-density polyethylene (LLDPE) composite using titanate-modified AlN. The composite exhibited good mechanical properties and a thermal conductivity of 1.0842 W m^−1^ k^−1^ with 30 vol.% of AlN.

In view of the aforementioned studies, PBS and its composites are expected to have a wide range of applications in the engineering industry. To our knowledge, the thermomechanical properties of PBS-filled AlN nanocomposites have never been investigated. In this study, we modified the surface of AlN with different treating agents. We also aimed to investigate the effects of incorporating a low faction of AlN on the physicomechanical properties of the PBS/AlN nanocomposite.

## 2. Materials and Methods

### 2.1. Materials

PBS (Solpol 5000J) was obtained from Gio Soltech Co. Ltd. (Gangwon-do, South Korea). Aluminum nitride (AlN), purchased from Sigma–Aldrich, was used as a thermally conductive filler. (3-Aminopropyl) triethoxysilane (APTES), vinyl-triethoxysilane (VTES), and polysilazane (PSZ) HTG1800 (Sigma–Aldrich, Seoul, South Korea) were used as surface-treating agents for AlN. All chemicals were used as received, with no further treatment.

### 2.2. Method

**Figure 1:**
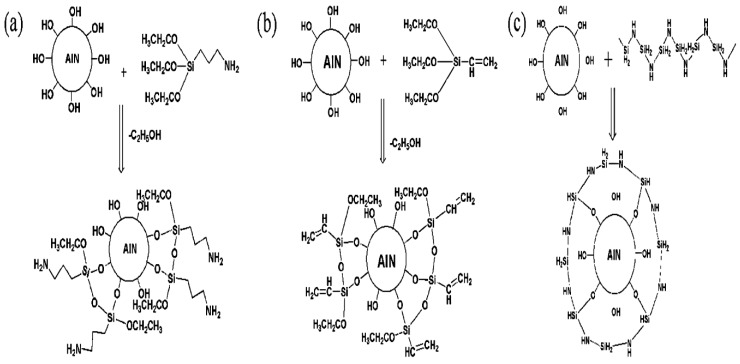
Schematic representation of the surface-treated AlN nanoparticles. (**a**) APTES-AlN, (**b**) VTES-AlN, and (**c**) PSZ-AlN.

The experimental study was performed in two phases. The first phase involved the modification of the surface of AlN to enhance its interaction with the PBS polymer matrix, which has a determining effect on the physicomechanical properties of the fabricated composite. The AlN nanoparticles were surface-functionalized with two silane coupling agents (APTES and VTES) and PSZ. The schematic representation of surface treated AlN nanoparticles is shown in Figure 1. The second phase involved the fabrication of PBS/AlN nanocomposites through the incorporation of the nanoparticles into the PBS matrix via a melt compounding process. The detailed experimental procedures are described below.

#### 2.2.1. Nanoparticle Treatment

The AlN nanoparticles were sonicated in ethanol for 30 min prior to salinization. Appropriate amounts of APTES and VTES (8–10 wt % based on the weight of AlN nanoparticles) were added to flasks containing ethanol and the mixtures were stirred at 65 °C for 5 h. Then, the sonicated AlN nanoparticles were added to each silane/ethanol solution under stirring for 2 h while the temperature was maintained at 65 °C. The final treated nanoparticles were separated through filtration. Finally, the nanoparticles were vacuum-dried at 80 °C to remove the remaining solvents, followed by oven drying at 80 °C until the AlN nanoparticles were completely dry. The AlN nanoparticles modified in this way were denoted as AlN-APTES and AlN-VTES, according to the silane coupling agent used for treatment.

PSZ surface treatment of the AlN nanoparticles was performed according to a previously reported procedure [22]. The AlN nanoparticles were first dipped and stirred in an excess amount of PSZ solution at room temperature for 1 h. Then, the mixture of AlN and the PSZ solution was filtered to remove the excess PSZ. The filtered PSZ-coated AlN nanoparticles were placed in a convection oven at 160 °C to allow moisture crosslinking of the PSZ on the surface of the nanoparticles. While drying, the PSZ-treated AlN was gently ground every 30 min for 2 h to prevent aggregation. Finally, the nanoparticles were left in the oven for 24 h. The AlN particles treated via this procedure were denoted as AlN-PSZ.

#### 2.2.2. Composite Fabrication

PBS/AlN nanocomposites were fabricated via melt compounding using a twin-screw extruder (model BA-11, Bau Tech, Seoul, Korea). Before the extrusion process, the PBS matrix was oven-dried at 80 °C for 24 h to remove surface moisture, which has the possibility of inducing void formation during the composite fabrication process. The PBS was mechanically mixed with 5 wt % of the nanoparticles and the mixture was fed to the extruder. The synthesized nanocomposites were collected from the nozzle and quenched with water. The nanocomposites synthesized using the extruder were pelletized and molded into different test samples using a mini-molder (model BA-915, Bau Tech). The PBS/AlN nanocomposites fabricated using raw AlN, APTES-AlN, VTES-AlN, and PSZ-AlN were denoted R-PBS, A-PBS, V-PBS, and P-PBS, respectively. Neat PBS was also prepared, as a control sample.

### 2.3. Characterizations

#### 2.3.1. Nanoparticle Characterization

Fourier transform infrared (FTIR, Nicolet iS5, Thermo Fisher Scientific, Seoul, Korea) spectroscopy and X-ray photoelectron spectroscopy (XPS, K-Alpha, Thermo Fisher Scientific) analyses were performed on the raw and modified AlN nanoparticles to study the binding interactions on the surface of the AlN particles before and after surface treatment. The FTIR analyses were performed in the wavelength range of 4000–400 cm^−1^ using KBr powder pellets. The XPS was performed at 1486.6 eV using Al Kα as the X-ray source. During the deconvolution of the XPS spectrum, the Gaussian-fitted peaks were kept constant.

#### 2.3.2. Composite Characterization

The cross-sectional morphologies of freeze-fractured samples of neat PBS and PBS/AlN nanocomposites were investigated using field-emission scanning electron microscopy (FE-SEM, Sigma, Carl Zeiss, Oberkochen, Germany). To make the samples conductive, platinum sputtering was performed on each sample prior to the FESEM observation. The melting and crystallization behavior of the neat PBS and its nanocomposites were investigated using differential scanning calorimetry (DSC, DSC-Evo, KEP Tech., Mougins, France) in a nitrogen atmosphere. The samples were heated to 150 °C from room temperature at a heating rate of 10 °C min^−1^. They were then cooled to room temperature and reheated to 150 °C at the same heating rate. The thermal property results were collected in the second heating cycle. Finally, the percentage crystallinity (*X_c_*) was calculated, as follows:

(1) $$X_{c}=\frac{∆H_{m}-∆H_{c}}{∆H_{m}^{o}\times w_{PBS}}$$

where *X_c_* represents the percentage of crystallinity of PBS; ∆*H_m_* and ∆*H_c_* are the enthalpies of melting and crystallization, respectively; $∆H_{m}^{o}$ is the enthalpy of fusion of 100% pure crystal PBS (105.3 Jg^−1^) [23]; and $w_{PBS}$ is the volume fraction of PBS in the composites.

The thermal degradation temperature of the neat PBS and PBS/AlN nanocomposites was studied using thermogravimetric analysis (TGA, TGA-2050, TA Instruments, New Castle, DE, USA). The TGA was performed by heating the samples to 600 °C from room temperature at a heating rate of 20 °C min^−1^ in a nitrogen atmosphere. The thermal conductivity (*κ*, W m^−1^ K^−1^) of the neat PBS and PBS/AlN nanocomposites was determined using the Flash method, as indicated by Equation (2). The thermal diffusivity (*δ*, mm^2^ s^−1^) was measured using a flash laser instrument (LFA 467, NETZSCH Instruments Co., Selb, Germany). The heat capacity (*C_p_*, J g^−1^ K^−1^) was determined using DSC at room temperature. The water displacement method was used to measure the density ($\rho_{comp}$, g cm^−3^) of each sample. Then, the thermal conductivity was calculated, as follows.

(2) $$\kappa =\delta \times \rho_{comp}\times C_{p}$$

The tensile properties of the molded dog-bone-shaped specimens were determined using a universal testing machine (UTM, model UTM-301, R&B Corp., Daejeon, Korea). The measurements were performed using a 100 kgf load at a rate of 5 mm min^−1^ at room temperature. The storage moduli of the samples were determined using dynamic mechanical analysis (DMA, Triton Tech., London, UK) in the temperature range of –60–110 °C at a frequency of 1 Hz.

## 3. Results and Discussion

### 3.1. Nanoparticle Analysis

**Figure 2:**
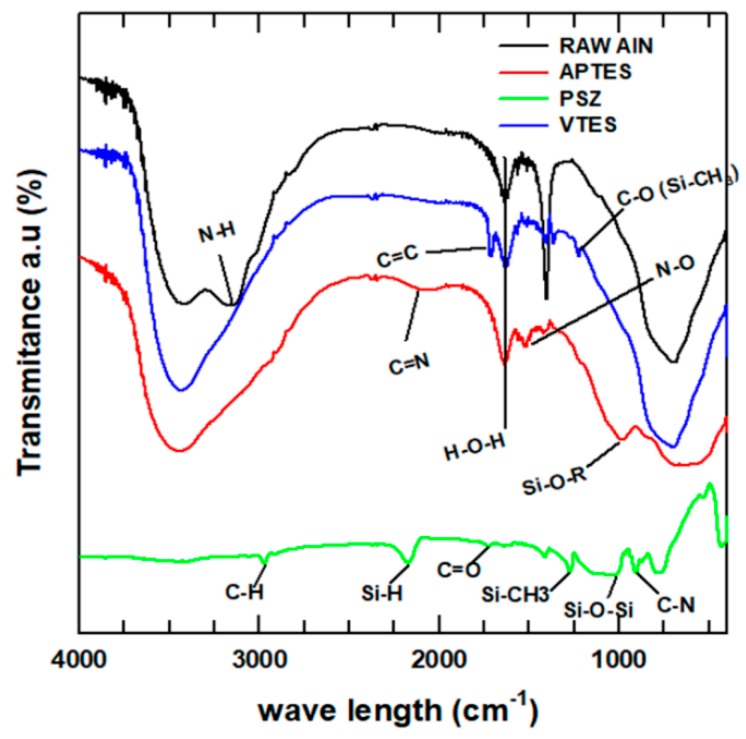
FTIR spectra of raw and treated AlN nanoparticles.

FTIR spectroscopy was performed to confirm the successful treatment of the AlN nanoparticles. The FTIR results for the raw and treated AlN nanoparticles are shown in Figure 2. The FTIR spectrum of the raw AlN has two peaks at, one at 3426 and one at 3170 cm^−1^, which are attributed to –OH and N–H binding forces, respectively. The spectral peaks at 1640 and 140 cm^−1^ are related to H–O–H and carbonates that arose from impurities on the surface of the AlN nanoparticles owing to exposure to the atmospheric air. For APTES-AlN, the nitrogen bonds in the APTES coupling agent are indicated by the peak at 2120 cm^−1^. In addition, the silane (Si–O–R) binding peak appeared at 990 cm^−1^. The unsaturated C=C binding in VTES appeared on the surface of VTES-AlN at 1715 cm^−1^. The presence of this unsaturated bond is expected to increase the reactivity of the AlN surface and enhance the interaction with the PBS matrix during the extrusion. However, a new peak appeared at 1220 cm^−1^, which is attributed to either C–O stretching or Si–CH_3_ stretching of the silane [24,25,26].

Compared with the two types of silane treated AlN nanoparticles, the IR spectrum of the PSZ-AlN was very different. Many new peaks appear for the surface of the treated nanoparticles. The peak at 1020 cm^−1^ is mainly related to the siloxane binding (Si–O–Si) of the polysiloxane. The other silicon-containing binding interactions represented by the peaks at 2165 and 1220 cm^−1^ were attributed to the stretching of the Si–H and Si–CH_3_ bonding, respectively [22,27,28]. In the FTIR spectroscopy graph, we observed that the hydrophilic parts of the raw AlN nanoparticles were reduced after the particle surface treatment.

**Figure 3:**
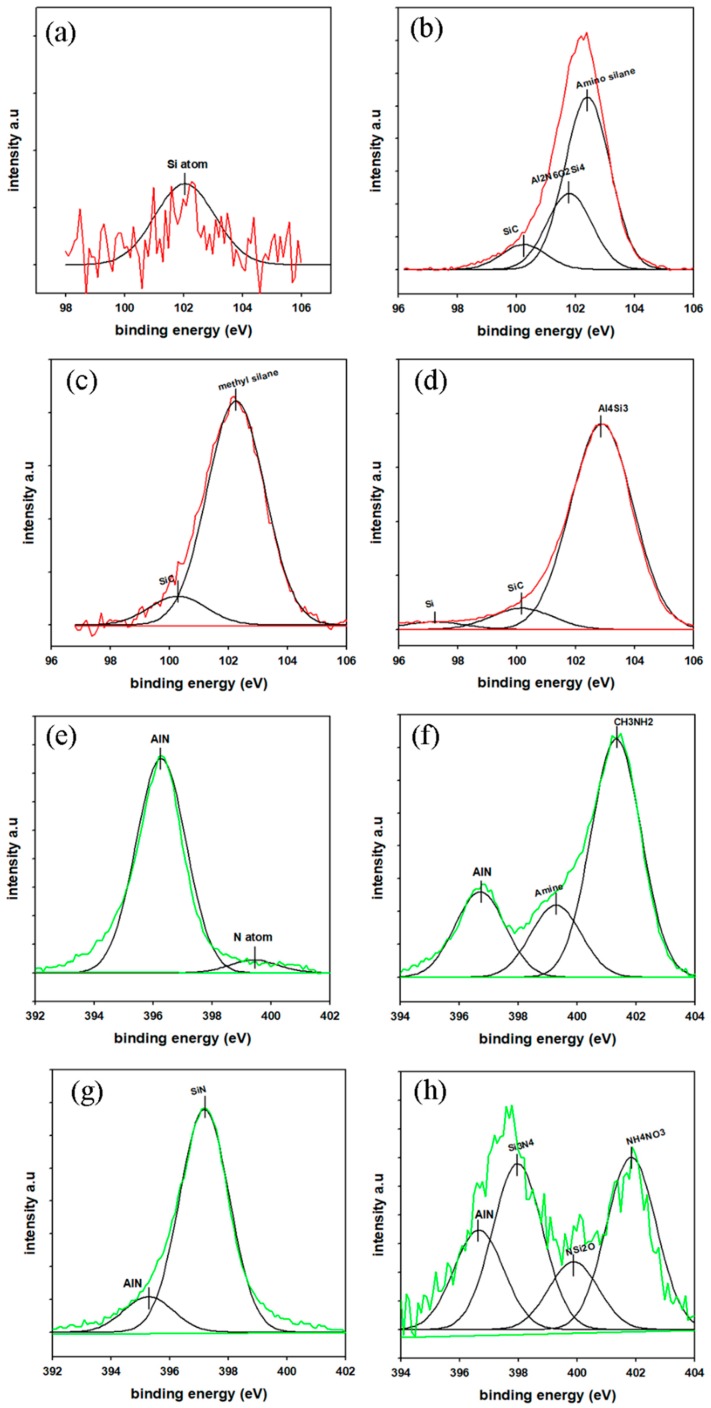
Si2p (top) and N1s (bottom) deconvoluted XPS spectrum of (**a**,**e**) raw AlN, (**b**,**f**) APTES-AlN, (**c**,**g**) VTES-AlN, and (**d**,**h**) PSZ-AlN.

In addition to the FTIR analysis, the elemental composition and binding energies of the raw and treated AlN nanoparticles were quantitatively analyzed using XPS. The XPS survey curve of the nanoparticles is presented in Figure S1. The figure shows the changes in the composition of the selected elements after the treatment of the AlN nanoparticles. The quantitative change of the atomic percentage composition is presented in Table S1. These variations of the elemental composition indicate the formation of new interactions and bonds after the treatment with the AlN nanoparticles. The N1s and Si2p spectra of the raw and treated AlN nanoparticles were deconvoluted to determine the specific type of bonds on the surface of the particles. The deconvoluted graphs are shown in Figure 3. The intensity of the AlN binding for N1s at 396.2 eV decreased after the particles underwent the surface modification. New binding peaks of N–H and H_3_C–NH_2_ appeared for APTES-AlN, indicating the formation of bonds between the surface of AlN and the APTES coupling agent. Similarly, there was a new peak at 397.2 eV, indicating the formation of Si-N bonds between the AlN surface and VTES. PSZ-AlN also exhibited new peaks at 398, 399.88, and 401.85 eV, which are attributed to Si_3_N_4_, NSi_2_O, and NH_4_NO_3_. On the other hand, the deconvoluted graph of Si2p, for the raw AlN only, has a peak for a small amount of silicon atoms found as an impurity on the surface of AlN. However, the Si-C binding peaks appeared at 100.25 eV after the treatment of the nanoparticles. The amino silane and methyl silane bonds arising from the APTES and VTES were represented by peaks at 102.4 and 102.25 eV for APTES-AlN and VTES-AlN, respectively. In general, the XPS analysis confirmed that the AlN particle treatment methods were performed successfully [22,27,28,29]. 

The amount of treating agents attached to the surface of the AlN particles was determined by heating the samples to 800 °C using TGA. The TGA curves for raw and treated AlN nanoparticles are shown in Figure S2. Most silane compounds degrade completely at 600 °C. Similarly, the APTES-AlN and VTES-AlN showed no mass change after 600 °C, because the coupling agents had already degraded. However, the PSZ-AlN particles were heated to 800 °C, owing to the high bond strength that is difficult to break at lower temperatures. According to the TGA curve, the silane-treated samples underwent a mass reduction of >15% after being heated at 600 °C. In contrast, the PSZ-AlN exhibited only a 7% mass reduction. This indicated that silane agents formed bonds and coated the surface of nanoparticles more easily than PSZ.

### 3.2. Nanocomposite Analysis

#### 3.2.1. Morphology

**Figure 4:**
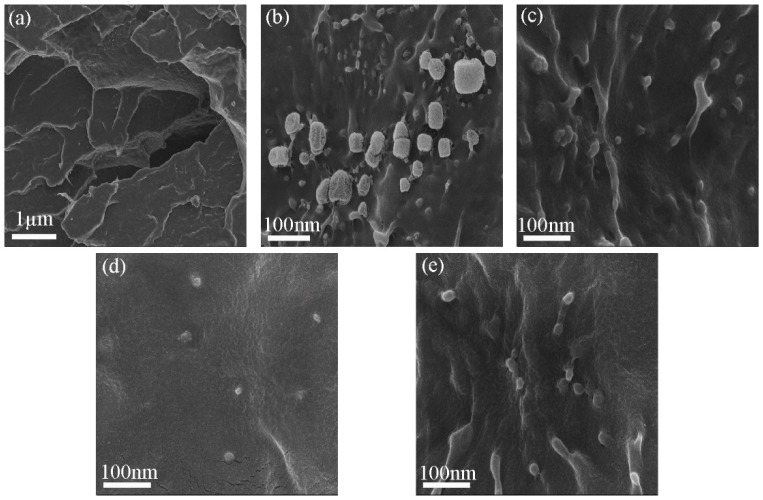
SEM image of freeze fractured samples (**a**) neat PBS, (**b**) R-PBS, (**c**) A-PBS, (**d**) V-PBS, and (**e**) P-PBS.

The morphology of the freeze-fractured cross-sectional surface of the PBS matrix before and after the incorporation of AlN nanoparticles was investigated using SEM. The SEM micrograph of the neat PBS and its composites are shown in Figure 4. The ductile behavior of the matrix gave the neat PNS a rough fractured surface. Additionally, voids and cracks formed owing to the poor mechanical properties of the matrix. After the inclusion of AlN nanoparticles, the fracture surface of the PBS/AlN nanocomposites became smoother, indicating the emergence of brittle behavior [30]. The AlN nanoparticles in the R-PBS composite were located very close to each other. The attractive interaction between the AlN nanoparticles in the R-PBS composite inhibited the uniform dispersion of the nanoparticles in the PBS matrix. This attractive force finally led to the formation of networks and aggregation between the AlN nanoparticles in the matrix [31]. The presence of aggregated nanoparticles was confirmed by measuring the nanoparticle size using SEM (Figure S3), which exceeded 100 nm. In addition, the AlN nanoparticles in R-PBS were exposed to the polymer surface, which indicated poor wettability between the AlN and the PBS matrix. On the other hand, the SEM images showed a reduction of the attractive force between the nanoparticles with the incorporation of surface-treated AlN into the PBS matrix. This indicated that the surface treatment of the nanoparticles had the possibility to change the surface chemistry of AlN, reducing the attractive force responsible for the formation of aggregates. As a result, the surface-treated AlN nanoparticles exhibited better adhesion and dispersion than the nanoparticles in R-PBS.

#### 3.2.2. Thermal Behaviors

**Figure 5:**
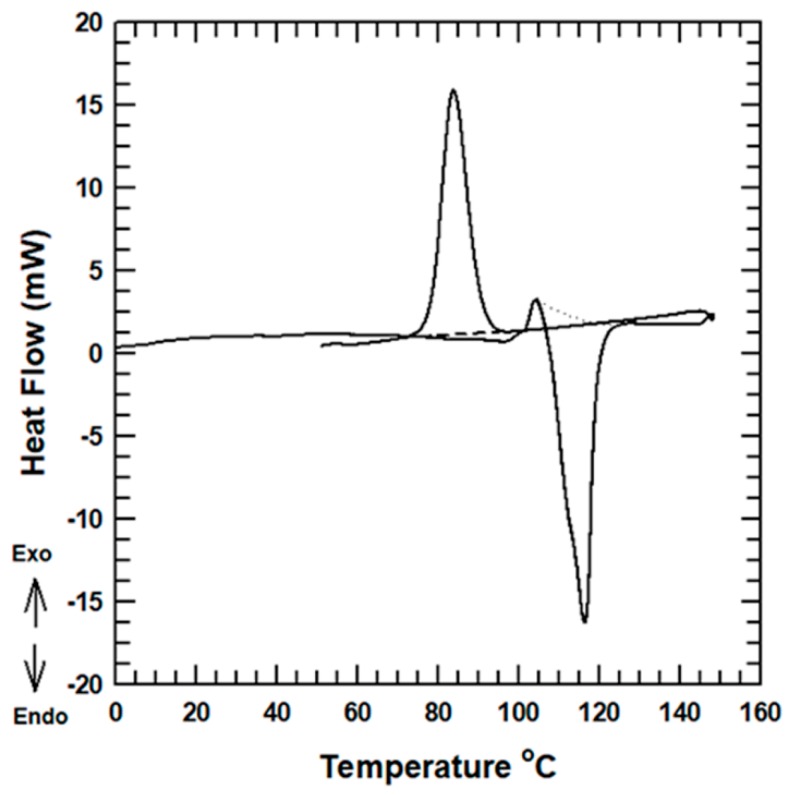
DSC thermograph curve of the neat PBS.

The melting and crystallization behavior of the neat PBS and its composites, observed during the second heating cycle, were studied using DSC. The DSC thermograph of the neat PBS sample is shown in Figure 5. The detailed melting and crystallization curves for the PBS nanocomposites are presented in Figure S4. PBS is a semi-crystalline and renewable polymer whose mechanical and morphological properties are affected by the level of crystallinity [32]. The change of the degree of crystallinity of PBS after the inclusion of AlN nanoparticles is presented in Table 1.

The melting point and crystallization point of the PBS/AlN composites did not show significant change compared with those of the neat PBS. The change of the melting and crystallization points did not differ by more than 1 °C between the tested samples. This showed that the AlN nanoparticles did not have a nucleating effect during the crystallization process of the matrix [33,34]. On the other hand, the degree of crystallinity varied with the addition of AlN nanoparticles. The degree of crystallinity of the PBS nanocomposites was lower than that of the neat PBS. This was mainly due to the formation of interfacial interaction between the PBS and the AlN nanoparticles. The difference in the level of crystallinity is expected to affect the tensile property of the samples.

#### 3.2.3. Thermogravimetry

**Figure 6:**
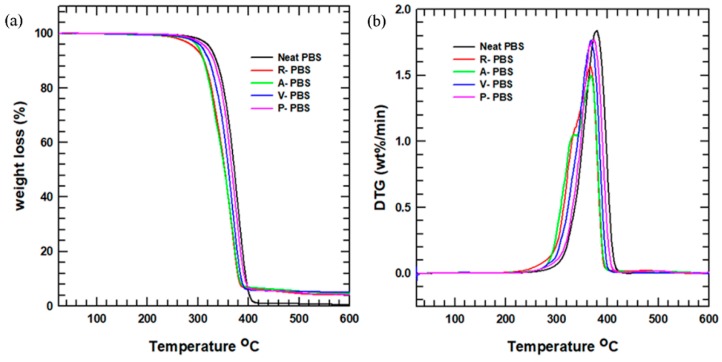
(**a**) TGA curve of the neat PBS and its composites; (**b**) Derivative thermogram curves of TGA.

The thermal degradation of the neat PBS and its composites was investigated using TGA. The weight loss (%) under TGA analysis indicated the successful fabrication of the PBS/AlN nanocomposites with 5% filler loading. The TGA curves for the samples are shown in Figure 6a. The curves show that the neat PBS had good thermal stability compared with its composites. The onset and offset degradation temperatures for each sample are presented in Table S2. The 5% and 95% weight loss in the matrix are considered the onset and offset degradation temperatures, respectively. The thermal stability was enhanced after the incorporation of surface-treated AlN, owing to the improvement of the interfacial interaction between the PBS matrix and the AlN nanofillers.

Likewise, the temperature at which the maximum degradation occurred is shown in Figure 6b, representing the derivative of the TGA curve as a function of temperature. This figure also demonstrates that the temperature range of thermal degradation differed for each sample, with the composite samples having a larger range than the neat PBS. This indicated that the rate of thermal degradation was reduced with the addition of AlN nanoparticles to the PBS matrix. This improvement of the thermal property makes the composites suitable for application in devices that operate under slow heating.

#### 3.2.4. Thermal Conductivity

**Figure 7:**
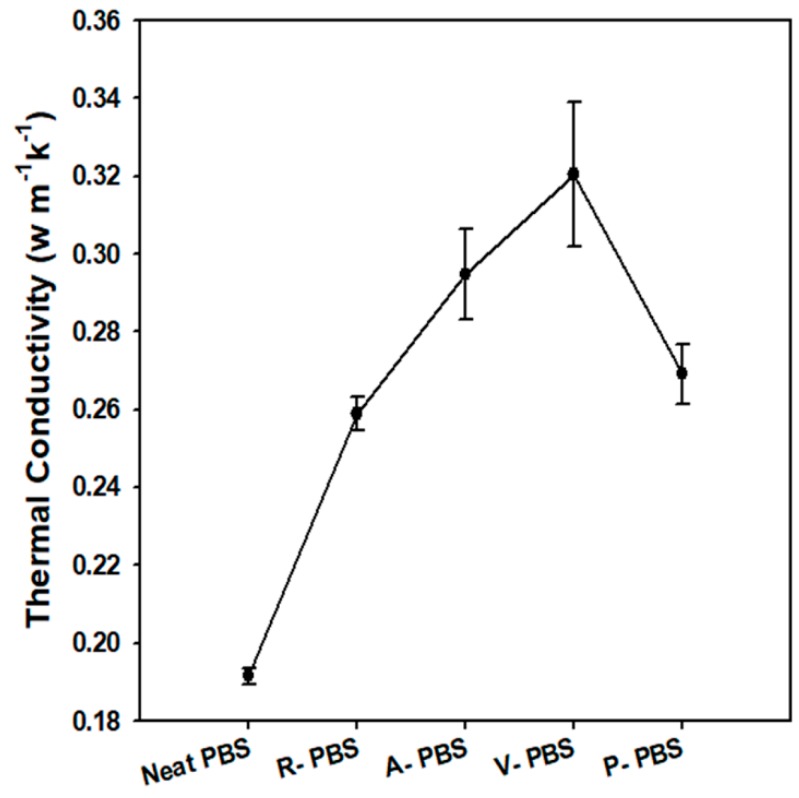
Thermal conductivity of neat PBS and its composites.

The thermal conductivity of the tested samples of neat PBS and its nanocomposites is shown in Figure 7. The thermal conductivity of a specimen measured using the flash laser method is dependent on the three parameters shown in equation 2. The thermal diffusivity and specific heat capacity of each sample is presented in Table S3. The specific heat capacity exhibited a small drop with the inclusion of AlN nanoparticles while the density of the nanocomposites became higher than the neat PBS. Therefore, the product of specific heat capacity and density was almost the same for each sample. Thus, the thermal conductivity of each sample was mainly dependent on the change in the thermal diffusivity of the corresponding sample. As expected, the thermal conductivity of the neat PBS was too low: <0.2 W m^−1^ k^−1^. The incorporation of the thermally conductive AlN nanoparticles increased the thermal conductivity of the fabricated PBS/AlN nanocomposites. Among the nanocomposites, V-PBS had the highest thermal conductivity, 67.3% higher than that of the neat PBS. Considering only 5 wt % AlN nanoparticles was added, the enhancement on thermal conductivity of the PBS composites is promising. This improvement of the thermal conductivity is attributed to two main factors. First, the incorporation of raw AlN into the matrix formed a network owing to the attractive force generated between the nanoparticles (Figure S3c). This caused the thermally conductive nanocomposites to transport heat more easily compared with the neat PBS [35]. The second factor was the improved interfacial interaction and dispersion of treated AlN nanoparticles (Figure S3b) [36]. For instance, the VTES-AlN-treated nanoparticles had more unsaturated (C=C) bonds on the surface than the raw AlN, which makes them more reactive to bond formation with the PBS matrix. As a result, these treated AlN nanoparticles formed good adhesion with the matrix. Generally, the inclusion of treated AlN nanoparticles improved the thermal conductivity of the nanocomposites.

#### 3.2.5. Tensile Property

**Figure 8:**
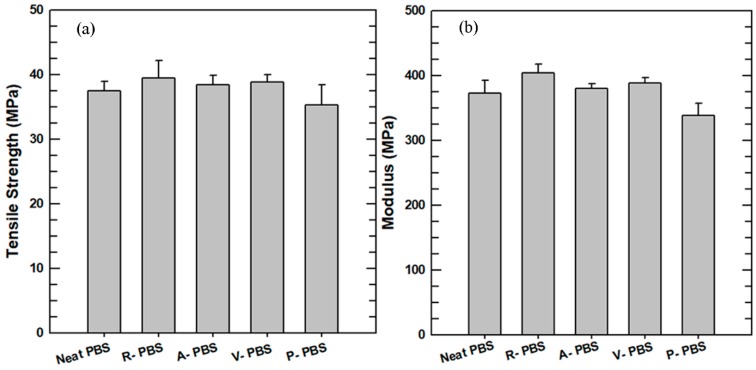
Tensile properties (**a**) tensile strength, and (**b**) Young’s modulus.

The tensile properties of the neat PBS and its composites were investigated using the UTM. The results for the tensile strength and Young’s modulus are presented in Figure 8. The tensile strength exhibits a small increase for R-PBS, A-PBS, and V-PBS compared with the neat PBS. Previous studies [37,38] showed that there is usually a decrease in the tensile strength of composites with the addition of ceramic fillers. However, in this study, the tensile strength of the PBS nanocomposites did not decrease with the incorporation of AlN. This result is mainly due to the change in the degree of crystallinity of the nanocomposites. The degree of crystallinity has an inverse effect on the tensile strength of the nanocomposites. As the level of crystallinity increases, the stress–strain transfer at the interface between the PBS and the AlN nanoparticles decreases. Among the samples investigated in this study, the neat PBS had the highest degree of crystallinity and lowest tensile strength.

The Young’s modulus exhibited the same trend as the tensile strength. The Young’s modulus of the R-PBS was 15–20 MPa higher than those of the A-PBS and V-PBS. This was attributed to the accumulation and aggregation of AlN nanoparticles on specific sites in the matrix, which increased the stiffness of the composites. On the other hand, after the surface-treated AlN nanoparticles were incorporated into the PBS matrix, the composites could no longer withstand the tensile load, as the particles were highly dispersed throughout the matrix. The reduction of the tensile strength and Young’s modulus of P-PBS is mainly associated with the formation of voids and cracks, despite the acceptable interaction between the PBS matrix and the PSZ-AlN nanoparticles.

#### 3.2.6. Dynamic Mechanical Property

**Figure 9:**
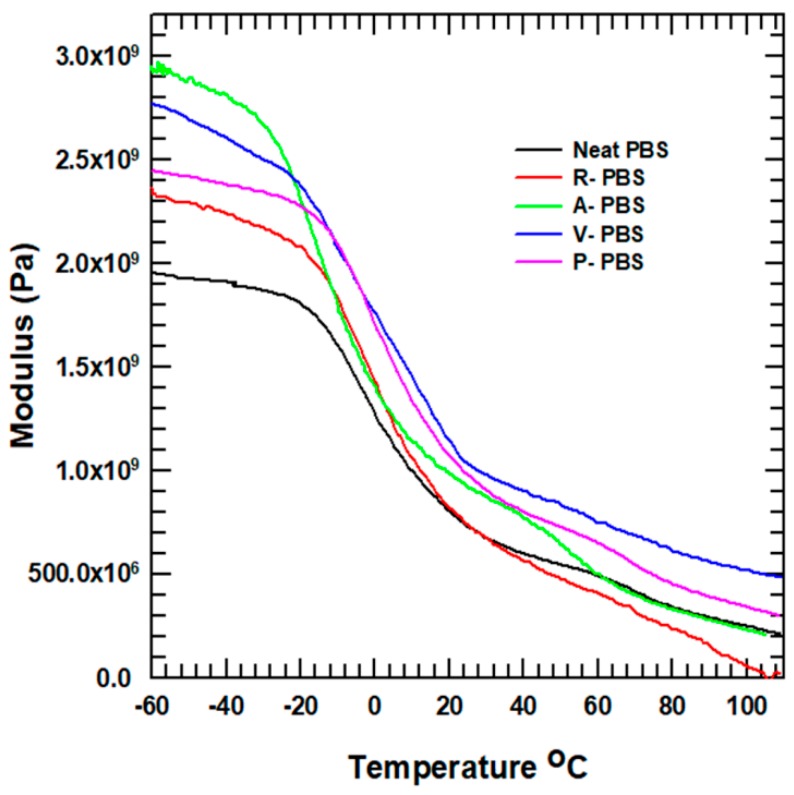
Storage modulus of the neat PBS and its composite.

The storage modulus as a function of temperature was measured using DMA from −60 °C to 110 °C. The transition from a glassy to rubber state, for each sample, is presented in Figure 9. The modulus exhibited improvement after the addition of AlN nanoparticles to the PBS matrix. At the starting temperature, the A-PBS exhibited improvement, 53.2% and 24.46% on the storage modulus compared with the neat PBS and R-PBS, respectively. Previous studies indicated that the storage modulus of a composite is influenced by the filler loading percentage rather than the interfacial adhesion between the filler and the matrix. However, this improvement in the modulus may be related to the homogeneous dispersion of APTES-AlN nanoparticles throughout the PBS matrix. Consequently, the distribution of nanoparticles in the composite enhanced the storage modulus, which is a measure of the stored energy.

## 4. Conclusions

PBS is a biodegradable material that is expected to replace many conventional polyolefins in various engineering fields. In this study, novel PBS/AlN nanocomposites were synthesized via melt compounding using twin screw extrusion. The AlN nanoparticles were synthesized with different treating agents. FTIR and XPS analysis of the nanoparticles confirmed that the surface modification was performed successfully. The FE-SEM morphology of the fabricated nanocomposites revealed that the treated nanoparticles had suitable interaction with the PBS matrix compared with the raw AlN. The inclusion of AlN nanoparticles did not change the crystallization or melting temperature of the PBS matrix. The thermal conductivity of the PBS composites exhibited an enhancement of >65% with the inclusion of 5% VTES-AlN in the PBS matrix. Additionally, the storage modulus was improved with the incorporation of treated AlN nanoparticles. This study shows that PBS/AlN nanocomposites have great potential for use in electronic and automotive engineering.

## Figures and Tables

**Table 1:** Crystallization and melting behavior of neat PBS and its composites.

Sample	Melting Pt. (°C)	Crystallization Pt. (°C)	∆H_m_	∆H_C_	X_C_ (%)
Neat PBS	116.395	83.9	96.46	81.83	13.9
R- PBS	116.1	84.98	71.716	63.26	8.45
A- PBS	116.16	84.39	79.38	69.202	10.17
V- PBS	116.275	83.975	86.6	76.92	9.67
P- PBS	116.263	84.8	73.85	66.57	7.278

## Supplementary Materials

The following are available online at http://www.mdpi.com/2073-4360/11/1/148/s1.
