# Supplementary material for: Surface Modification of Aluminum Nitride to Fabricate Thermally Conductive poly(Butylene Succinate) Nanocomposite
Source: Polymers (Basel). 2019 Jan 16;11(1):148. doi: 10.3390/polym11010148 (PMC6401904; doi:10.3390/polym11010148)
Supplement: Supplementary file 1 [file polymers-11-00148-s001.pdf]

supplemenatry material

Surface modification of aluminum nitride to fabricate thermally conductive poly(butylene succinate) nanocomposite

Zelalem Lule and Jooheon Kim*

School of Chemical Engineering & Materials Science,

Chung-Ang University, Seoul 156-756, Republic of Korea

*Corresponding author: Jooheon Kim, Tel:+82-2-820-5763; Fax:+82-2-812-3495; E-mail address:

jooheonkim@cau.ac.kr (J. Kim)

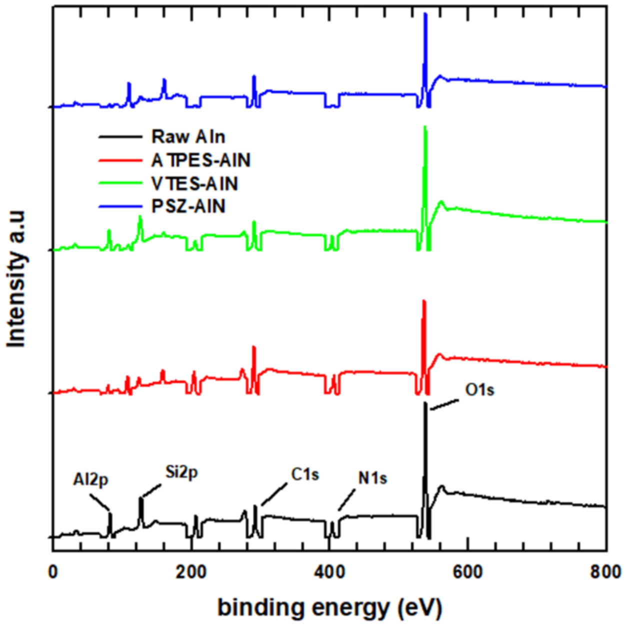

Figure S1. XPS survey curve of Raw and treated AlN nanoparticles.

Table S1. The atomic percentage of Raw and treated AlN nanoparticles.

Sample	Atomic Percentage (%)				
	O1s	C1s	N1s	Si2s	Al2p
Raw AlN	44.06	21.95	6.19	1.15	26.65
APTES-AlN	29.2	39.59	10.18	11.54	9.5
VTES-AlN	43.23	21.86	6.45	3.78	24.68
PSZ-AlN	38.69	30.24	1.65	24.39	5.03

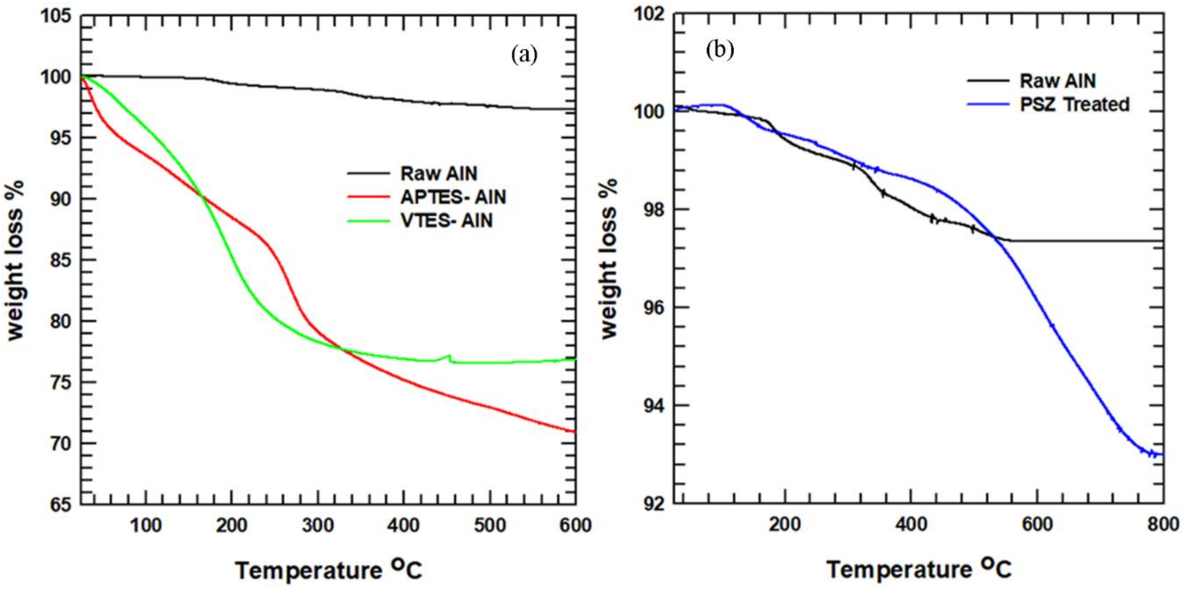

Figure S2. TGA curve of the raw and treated AlN nanoparticles.

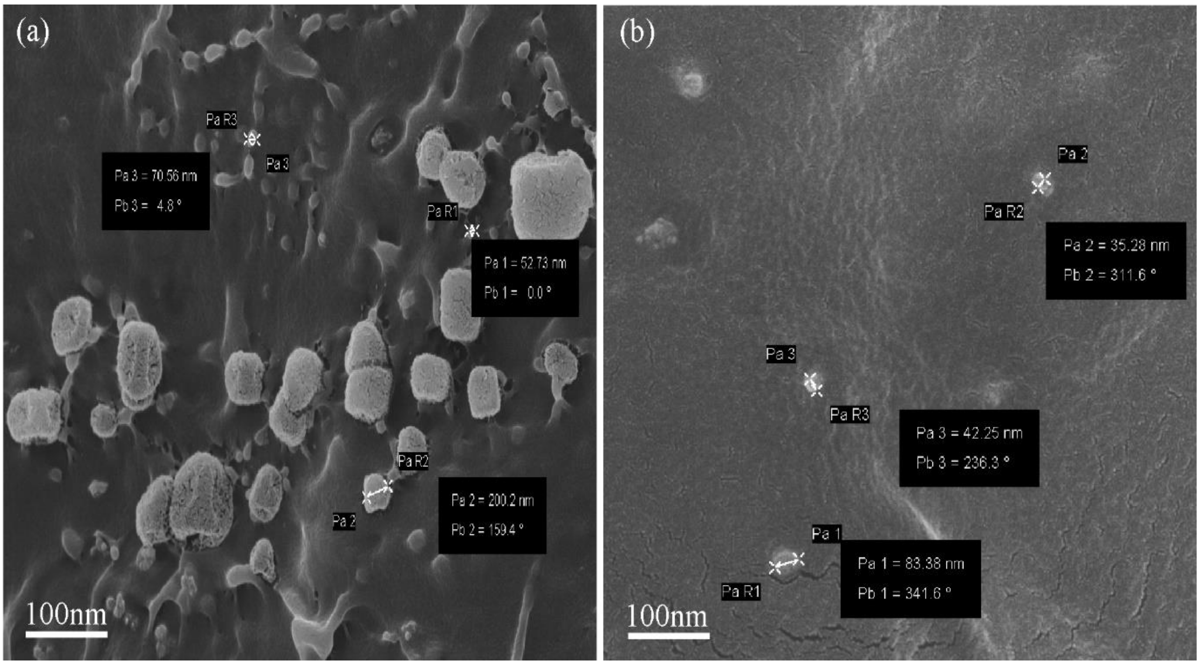

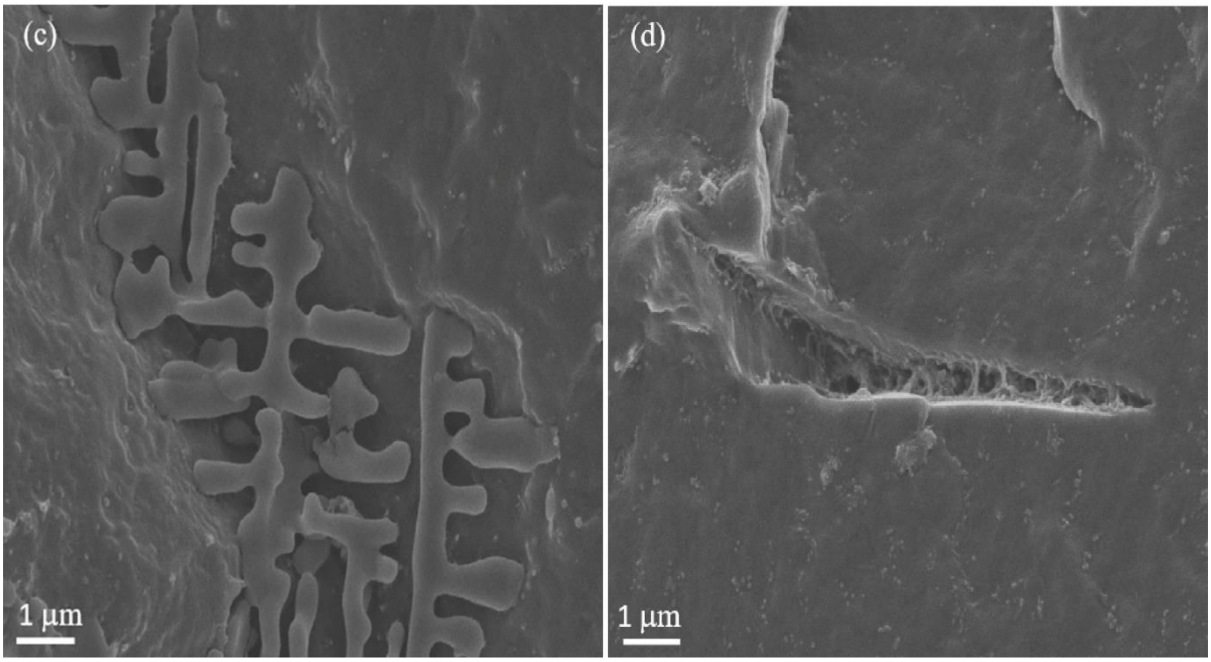

Figure S3. SEM image with size distribution and dispersion of AlN nanoparticles (a), (c) R- PBS, (b) V- PBS, and (d) P- PBS.

Table S2. Thermal degradation temperatures of Neat PBS and its composites.

Sample	On-set T_d (ºC)	Off-set T_d (ºC)	Max. degradation T_d (ºC)
Neat PBS	323.68	397.43	379.89
R- PBS	292.77	383.89	366.36
A- PBS	300.98	385.75	368.33
V- PBS	307.96	387.42	368.1
P- PBS	315.78	396.6	374.2

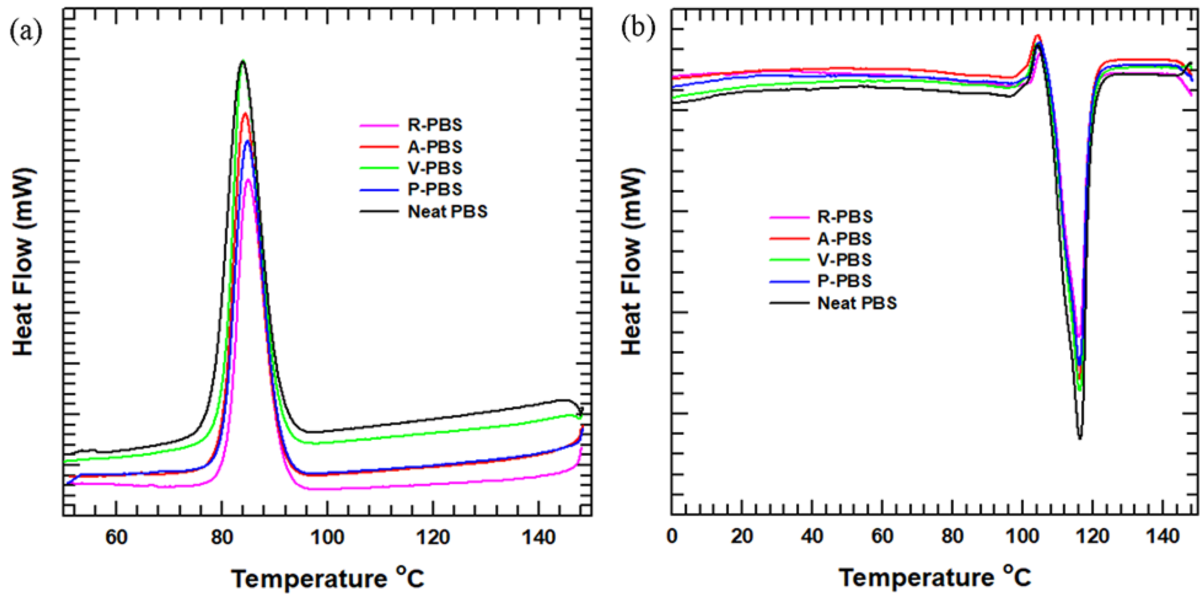

Figure S4. DSC thermograph of the Neat PBS and composites; (a) crystallization curve, and (b) melting curve.

Table S3. Thermal diffusivity and heat capacity of neat PBS and PBS/AlN composites.

Sample	Thermal diffusivity, δ ($mm^2\ s^{-1}$)	Heat capacity, Cp ($J\ g^{-1}\ K^{-1}$)
PBS	0.0947±0.00198	1.6
R-PBS	0.101±0.00444	1.55
A- PBS	0.115±0.01174	1.55
V- PBS	0.125±0.01848	1.55
P- PBS	0.105±0.00769	1.55
